# Supplementary material for: Yiai Fuzheng decoction inhibits triple-negative breast cancer by remodeling the immune microenvironment
Source: Front Immunol. 2025 Sep 30;16:1615631. doi: 10.3389/fimmu.2025.1615631 (PMC12518410; doi:10.3389/fimmu.2025.1615631)
Supplement: Supplementary file 2 [file DataSheet1.zip › Supplementary File 2/Treatment/C_vs_D/07_Pathway_Analysis/Pathway_Analysis/Sig_Pathways_ID/Tryptophan metabolism.pdf]

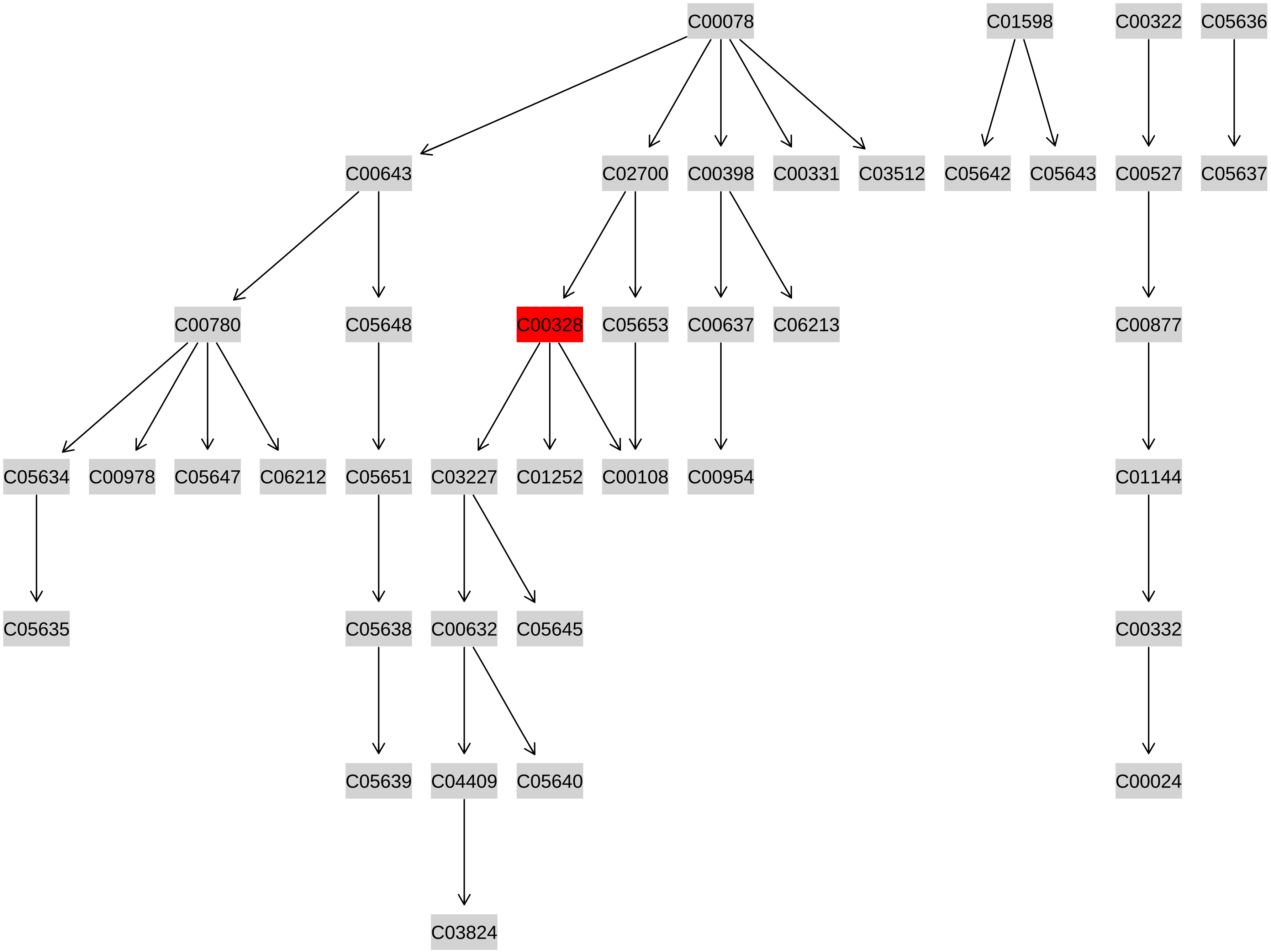
C00078
C01598
C00322
C05636
C00643
C02700
C00398
C00331
C03512
C05642
C05643
C00527
C05637
C00780
C05648
C00328
C05653
C00637
C06213
C00877
C05634
C00978
C05647
C06212
C05651
C03227
C01252
C00108
C00954
C01144
C05635
C05638
C00632
C05645
C00332
C05639
C04409
C05640
C00024
C03824
